# Bioluminescence Imaging of β Cells and Intrahepatic *Insulin* Gene Activity under Normal and Pathological Conditions

**DOI:** 10.1371/journal.pone.0060411

**Published:** 2013-04-04

**Authors:** Tokio Katsumata, Hisashi Oishi, Yukari Sekiguchi, Haruka Nagasaki, Dhouha Daassi, Pei-Han Tai, Masatsugu Ema, Takashi Kudo, Satoru Takahashi

**Affiliations:** Department of Anatomy and Embryology, Faculty of Medicine, University of Tsukuba, Tennodai, Tsukuba, Ibaraki, Japan; Baylor College of Medicine, United States of America

## Abstract

In diabetes research, bioluminescence imaging (BLI) has been applied in studies of β-cell impairment, development, and islet transplantation. To develop a mouse model that enables noninvasive imaging of β cells, we generated a bacterial artificial chromosome (BAC) transgenic mouse in which a mouse 200-kbp genomic fragment comprising the *insulin I* gene drives luciferase expression (*Ins1-luc* BAC transgenic mouse). BLI of mice was performed using the IVIS Spectrum system after intraperitoneal injection of luciferin, and the bioluminescence signal from the pancreatic region analyzed. When compared with MIP-Luc-VU mice [FVB/N-Tg(*Ins1-luc*)VUPwrs/J] expressing luciferase under the control of the 9.2-kbp mouse *insulin I* promoter (MIP), the bioluminescence emission from *Ins1-luc* BAC transgenic mice was enhanced approximately 4-fold. Streptozotocin-treated *Ins1-luc* BAC transgenic mice developed severe diabetes concomitant with a sharp decline in the BLI signal intensity in the pancreas. Conversely, mice fed a high-fat diet for 8 weeks showed an increase in the signal, reflecting a decrease or increase in the β-cell mass. Although the bioluminescence intensity of the islets correlated well with the number of isolated islets *in vitro*, the intensity obtained from a living mouse *in vivo* did not necessarily reflect an absolute quantification of the β-cell mass under pathological conditions. On the other hand, adenovirus-mediated gene transduction of β-cell-related transcription factors in *Ins1-luc* BAC transgenic mice generated luminescence from the hepatic region for more than 1 week. These results demonstrate that BLI in *Ins1-luc* BAC transgenic mice provides a noninvasive method of imaging islet β cells and extrapancreatic activity of the *insulin* gene in the liver under normal and pathological conditions.

## Introduction

In type I diabetes, autoimmune reaction to β cells leads to destruction of insulin-producing cells, and in type II diabetes, cumulative cell damage evoked by various stresses induces β-cell dysfunction, eventually resulting in insufficient insulin supply and a reduction in β-cell mass [1], [2]. Accurate assessment of β-cell mass is considered necessary for understanding both the pathogenesis and the prognosis of diabetes [3]. In human studies, various modalities such as positron emission tomography (PET), single photon emission computed tomography (SPECT), and magnetic resonance imaging (MRI) have been shown to be useful means for quantification of native and transplanted β-cell mass [4]. In contrast to other modalities, bioluminescence imaging (BLI) additionally provides quantifiable data with high throughput and inherently low background; however, it is difficult to use current BLI technology for *in situ* quantification of human β cells because the light emission quickly diminishes as it propagates through tissues [5]. In experimental animals, an increasing number of studies have proposed successful quantification of β-cell mass using BLI of mice expressing β-cell-specific reporters [6]–[8]. BLI has also been applied in animal studies on β-cell development, islet transplantation, and β-cell function [4], [8]–[11].

β-cell mass flexibly adapts to insulin requirement as shown by the fact that adult β cells expand in response to states of increased demand for insulin such as obesity and pregnancy [12], [13]. These dynamic changes in β-cell mass are probably controlled by a balance between programmed cell death and replication of existing β cells and/or neogenesis from precursor cells [13], [14]. To address the imbalance between these conditions in diabetes, development of novel β-cell treatment is necessary. In addition to islet-cell transfer from donors, insulin-producing cells from embryonic stem (ES) cells, inducible pluripotent stem cells, pancreatic exocrine cells, pancreatic duct cells, and hepatic oval cells could be directed to become insulin-producing cells [15]–[21]. However, most insulin-producing cells generated from other cell types did not achieve complete physiological actions such as glucose sensing and adequate insulin production that are performed by mature β cells. Indeed, recent analyses of human ES cell-derived insulin-producing cells revealed that the cells were often multihormonal and had gene expression profiles resembling immature endocrine cells [22].

In this study, we aimed to generate mice expressing a β-cell-specific reporter with a more intense luminescence and a lower background. For this objective, the bacterial artificial chromosome (BAC) transgenesis was applied. BAC inserts are large (100–300 kb) and therefore carry almost all the regulatory sequences necessary for temporally and spatially correct expression that closely reflect endogenous gene activity independent of the genomic integration site [23], [24]. In addition, the *luc2* gene that is adapted for mammalian expression was used as a luminescent reporter to improve sensitivity. Here, we show that novel *Ins1-luc* BAC transgenic mice are useful for visualization of islet β cells and intrahepatic *insulin* gene activity under normal and pathological conditions.

## Materials and Methods

### Animals

**Figure 1:**
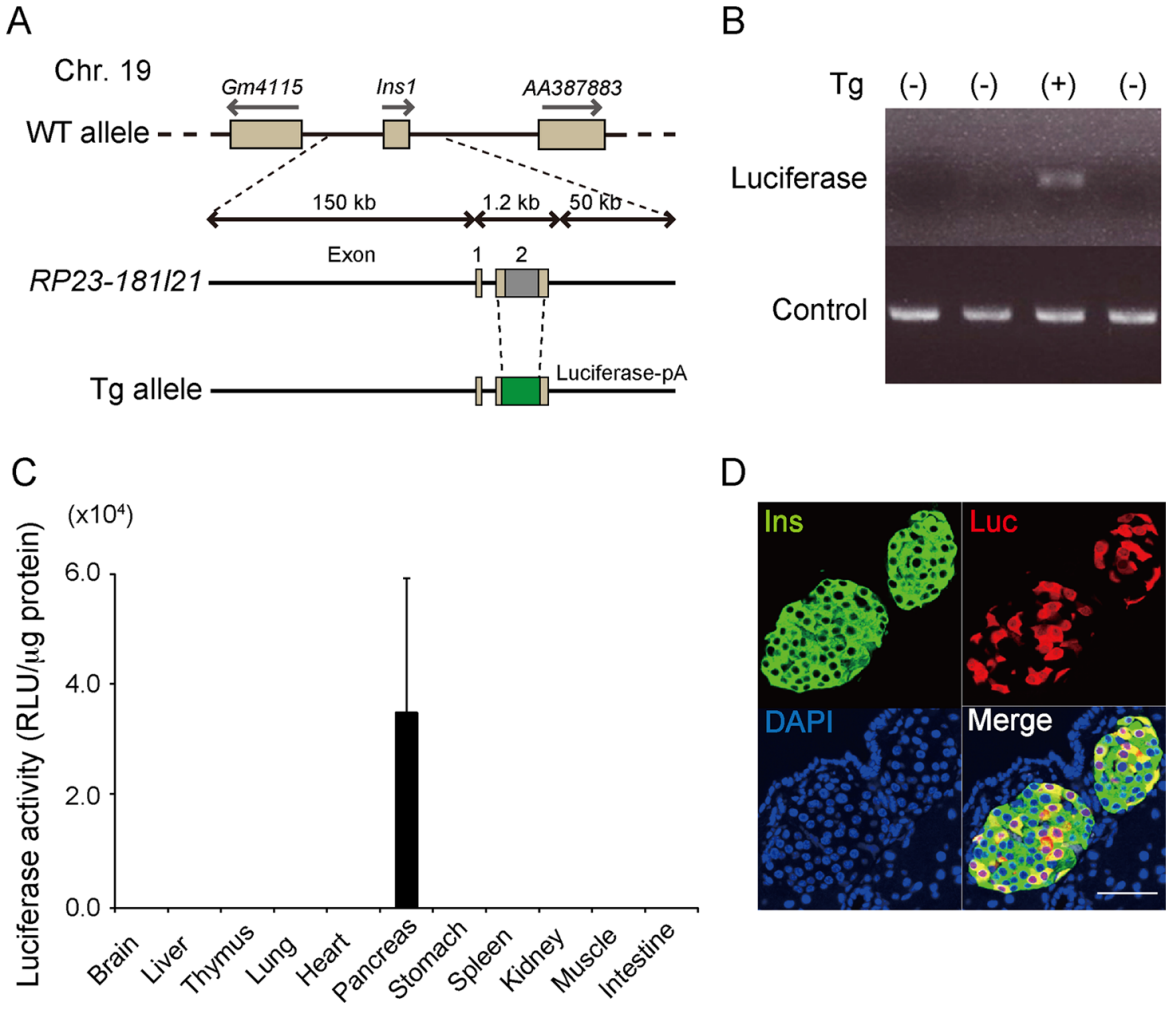
Generation of *Ins1-luc* BAC transgenic mice. (A) Diagrammatic representation of the transgene. (B) Representative example of a PCR-positive individual for genotyping. Control: interleukin-2. (C) Luciferase activity in tissue lysates of male *Ins1-luc* BAC transgenic mice at 4 to 6 weeks of age (n = 3). RLU: relative light unit. (D) Tissue sections of *Ins1-luc* BAC transgenic mouse stained with anti-insulin antibody (Ins), anti-luciferase antibody (Luc), and diamidino-2-phenylindole (DAPI). Scale bars: 50 µm.

All experiments were performed in compliance with the relevant Japanese and institutional laws and guidelines and approved by the University of Tsukuba animal ethics committee (authorization number 12–189). A luciferase gene fragment with the polyadenylation signal of human growth hormone was obtained by digestion of the pGL4.10 vector (Promega, Madison, WI, USA) with XhoI/BamHI. The *insulin I* gene in the BAC clone *RP23-181I21* (Invitrogen, Carlsbad, CA, USA), was replaced with the firefly luciferase gene using a Red/ET recombination system (Gene Bridges, Heidelberg, Germany) (Figure 1A). Recombinant BAC DNA linearized by PI-SceI digestion was used for pronuclear injection of fertilized eggs collected from ICR females. The injected eggs were transplanted into pseudopregnant ICR females. Transgenic mice expressing luciferase under the control of the mouse *Ins1* promoter [FVB/N-Tg(*Ins1-luc*)VUPwrs/J; Stock number: 007800; MIP-Luc-VU] were purchased from the Jackson Laboratory (Bar Harbor, ME, USA). Both lines of mice were continuously bred with the Jcl:ICR strain (Clea Japan, Tokyo, Japan).

### Screening of *Ins1-luc* BAC transgenic mice and determination of the transgene copy number

The genotype and copy number of the transgene were determined by means of regular PCR and quantitative PCR of the tail DNA, respectively [25]. The primer sequences for the luciferase gene were 5′-gagcagctgcacaaagccatg-3′ and 5′-cgctcatctcgaagtactcgg-3′ and for the control (*interleukin-2*), 5′-ctaggccacagaattgaaagatct-3′ and 5′-gtaggtggaaattctagcatcatcc-3′ [25].

### Measurement of luciferase activity

A luciferase assay kit (Promega) and Glomax 20/20 luminometer (Promega) were used to measure luciferase activity, which was expressed as relative light units (RLU) per milligram of protein. The protein concentration was determined using a Coomassie Protein Assay kit (Thermo Fisher Scientific, Waltham, MA, USA).

### Bioluminescence imaging

To detect the bioluminescence of free-fed *Ins1-luc* BAC transgenic mice and of MIP-Luc-VU mice using an IVIS spectrum (Caliper Life Sciences, Hopkinton, MA, USA), D-luciferin (5 mg/kg body weight, Promega) was injected intraperitoneally (IP) and imaged 5 and 10 minutes later, respectively. Luminescence images were captured with an integration time of 1 minute, and isometric regions of interest (ROIs) were drawn over the location corresponding to the pancreas for the quantification using Living Image software (Xenogen Corporation, Alameda, CA, USA). For studies in which BLI was performed *in vitro*, a variable number of equal-sized islets isolated from both strains were cultured overnight in RPMI with 10% FBS and 16.7 mM glucose and imaged as described elsewhere [8].

### Histological analysis

WT and *Ins1-luc* BAC transgenic mice were euthanized at 8 weeks of age, and the pancreatic tissues removed. Tissues were fixed in 10% formalin and embedded in paraffin. Tissue sections were incubated with guinea pig anti-insulin antibody (Abcam, Cambridge, UK) and rabbit anti-glucagon antibody (Linco Research, St. Charles, MO, USA) for 8 hours at 4°C. The antigens were visualized using appropriate secondary antibodies conjugated with alexa488 and alexa594 with nuclear staining using diamidino-2-phenylindole (DAPI) (Invitrogen). For measurement of β-cell mass, the removed pancreatic fragments were immediately weighed. Ten consecutive 5-µm sections 200 µm apart spanning the entire pancreas were stained with anti-insulin antibody. Images of the sections were scanned and analyzed using a Biorevo BZ-9000 microscope (Keyence, Osaka, Japan) and BZ-II analyzer software (Keyence). The relative areas stained for insulin were measured and multiplied by the pancreas weight to estimate the β-cell mass.

### Diabetes induction

*Ins1-luc* BAC transgenic mice were rendered diabetic at 6 weeks of age by an IP injection of streptozotocin (STZ, Sigma) at a dose of 200 mg/kg body weight in 0.1 M citrate buffer (pH 4.5).

### High-fat Diet

*Ins1-luc* BAC transgenic mice were fed either a control regular diet (RD) or a high-fat diet (HFD) consisting of 62.2% fat, 19.6% carbohydrate, and 18.2% protein content on a caloric basis (Oriental Yeast, Tokyo, Japan). To assess the effect of the HFD on glucose homeostasis, the body fat composition and fasting blood glucose (FBG) levels of the mice were examined by means of a LaTheta computed tomography (CT) system (Hitachi Aloka Medical, Tokyo, Japan) and a Drichem 3500 (Fujifilm, Tokyo, Japan), respectively. Bioluminescence images were collected every 4 weeks beginning when the mice were 6 weeks of age.

### Preparation of recombinant adenovirus vectors

Recombinant adenoviruses expressing mouse *Pdx1*, *NeuroD* (kindly gifted by Dr S. Yoshida), and *MafA* were prepared using a ViraPower Adenoviral Gateway Expression Kit (Invitrogen) [26]. In brief, cDNA fragments were cloned into a pENTR4 entry vector. To create expression clones and produce recombinant adenoviruses, the pENTR4 inserts were transferred into the pAd/CMV/V5-DEST destination vector using the LR recombination reaction, and PacI-linearized plasmids were transfected into 293A cells (Invitrogen) using Fugene 6 transfection reagent (Roche Diagnostics, Basel, Switzerland). Adenoviral constructs (5.0×10^9^ pfu/mouse) were injected via the tail vein, and serial BLI was performed before and after infection on designated days. On day 0, the initial BLI was performed before the viral infection.

### Statistical analysis

Data were expressed as the means ± standard errors of the means and compared using an unpaired *t* test. Probability values of less than 0.05 were considered significant.

## Results

We first obtained 68 founder (F0) mice. Of those 68 mice, 8 were transgenic, as confirmed by luciferase gene integration identified by PCR. On BLI screening of the 8 F0 transgenic mice, 4 mice did not emit any BLI signal, 2 mice exhibited an ectopic bioluminescence signal, and the remaining 2 mice exhibited considerable signal intensities emanating from the pancreatic region. Finally, germ line transmission was confirmed by transgene-specific PCR in 1 of the remaining 2 mice (*Ins1-luc* BAC transgenic mouse). For further experiments, the pups from *Ins1-luc* BAC transgenic mice and WT mice were identified by transgene-specific PCR (Figure 1B). To examine which organs expressed the reporter gene, different organ tissues at 6 weeks of age were dissected, and the luciferase activities determined. Luciferase activity was detected in the pancreatic extracts (3.5±2.4×10^4^ RLU/µg protein; n = 3), but not in the other tissue extracts including those from the thymus and pituitary glands, indicating that the reporter gene was expressed only in the pancreas (Figure 1C). Further immunohistochemical analysis using anti-insulin and anti-luciferase antibodies demonstrated that luciferase-expressing cells were colocalized only with the insulin-positive cells in the pancreas; however, only 11.9±5.2% (n = 3) of the insulin-expressing cells expressed luciferase (Figure 1D). Treatment of islets isolated from *Ins1-Luc* BAC transgenic mice with a proteasome inhibitor, MG132, resulted in coexpression of luciferase by most of the insulin-positive cells, suggesting that the relatively low frequency of the expression of the reporter in β cells depends mostly on the proteasomal degradation of luciferase (Figure S1). Using quantitative PCR analyses of genomic DNA isolated from MIP-Luc-VU and *Ins1-luc* BAC transgenic mice, the transgene copy number of the *Ins1-luc* BAC transgenic mice was determined to be 3, the same as that of the MIP-Luc-VU mice (data not shown) [8].

**Figure 2:**
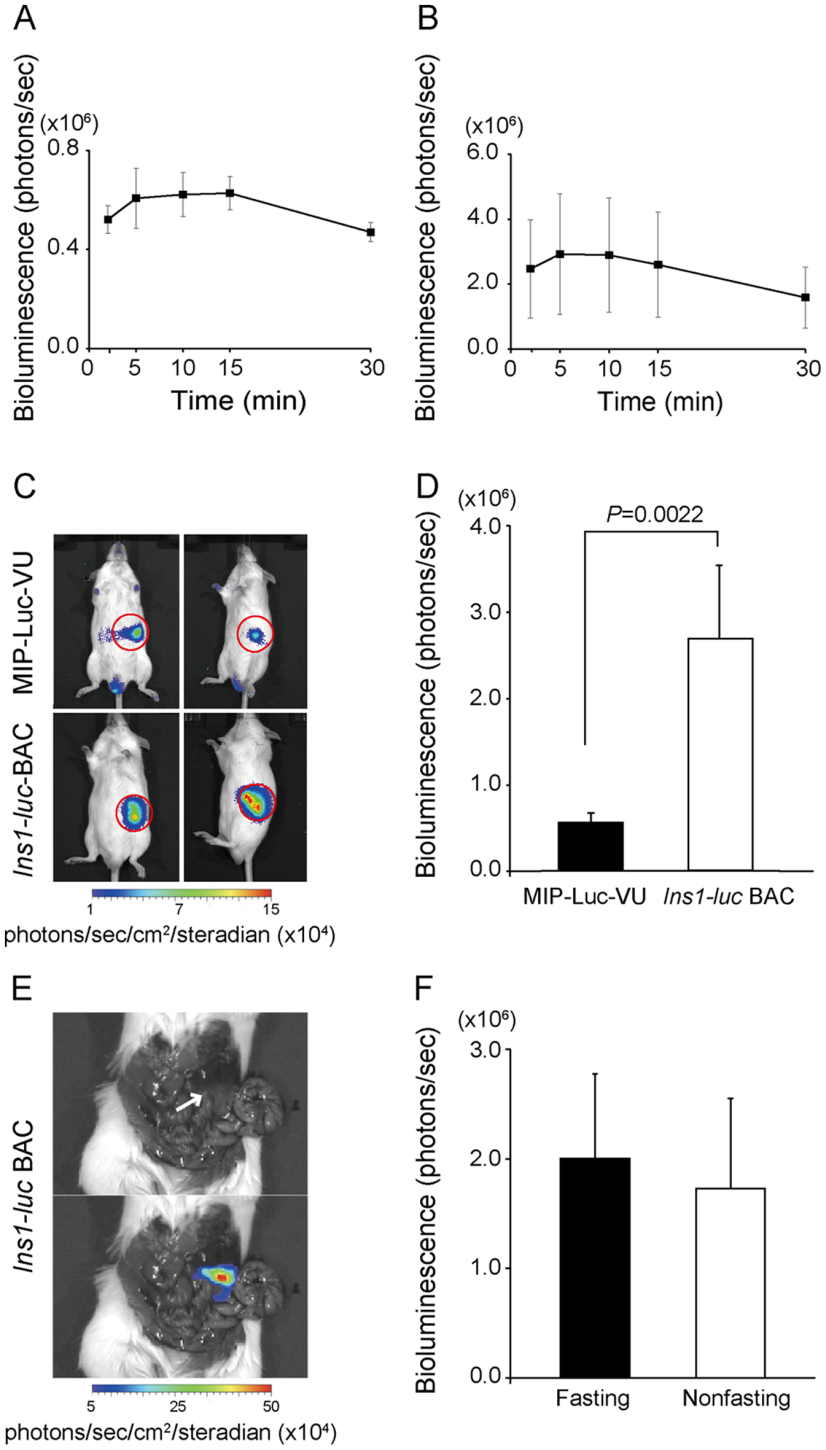
*In vivo* bioluminescence imaging of MIP-Luc-VU and *Ins1-luc* BAC transgenic mice. (A, B) Changes in bioluminescence intensity of (A) MIP-Luc-VU (n = 3) and (B) *Ins1-luc* BAC transgenic (n = 3) mice following intraperitoneal injection of luciferin. (C) Representative bioluminescence imaging of MIP-Luc-VU mice (upper) and *Ins1-luc* BAC transgenic mice (lower). Circles indicate regions of interest. (D) Quantification of signal intensity in male MIP-Luc-VU mice (n = 6) and male *Ins1-luc* BAC transgenic mice (n = 18). (E) Bioluminesence images of laparotomized *Ins1-luc* BAC transgenic mice immediately after injection of luciferin. The arrow indicates the pancreas. (F) Quantification of the signal intensity of *Ins1-luc* BAC transgenic mice in the fasting and nonfasting states.

Quantitation of bioluminescence *in vivo* critically depends on substrate availability and light-emission kinetics. To determine the kinetics of bioluminescence, MIP-Luc-VU and *Ins1-luc* BAC transgenic mice were imaged 2.5, 5, 10, 15, and 30 minutes after luciferin injection (5 mg/kg body weight, IP). Consistent with previous findings, levels of bioluminescence in MIP-Luc-VU mice peaked at approximately 10 minutes [8], whereas in *Ins1-luc* BAC transgenic mice, they peaked at 5 minutes (Figure 2A, B). Comparative measurements of the peak BLI signals of the pancreatic regions at 6 weeks of age revealed that the intensity in the *Ins1-luc* BAC transgenic mice (2.7±0.84×10^6^ photons/sec; n = 18) was significantly enhanced, by approximately 4-fold that of the MIP-Luc-VU mice (5.6±0.81×10^5^ photons/sec; n = 6; *P* = 0.022) (Figure 2C, D). As shown by laparotomy, the luminescence of the *Ins1-luc* BAC transgenic mice emanated only from the pancreas (Figure 2E). To examine the effect of overnight fasting on BLI, the BLI signals under the fasting and fed states of the same set of male *Ins1-luc* BAC transgenic mice (n = 5) were imaged at 3-day intervals. The intensity of the BLI signal under the fasting state (2.0±0.77×10^6^ photons/sec) did not differ from that under the fed state (1.73±0.82×10^6^ photons/sec; *P* = 0.814) (Figure 2F). Furthermore, the intensity of the BLI signal emitted in females (2.59±1.3×10^6^ photons/sec; n = 15) did not differ from that emitted in males (5.41±1.3×10^6^ photons/sec; n = 19) at 8 weeks of age (*P* = 0.13). Although signal attenuation in RIP-Luc mice over generations was reported because of a genomic imprinting of the transgene [27], we did not find the phenomenon in *Ins1-luc* BAC transgenic mice over 3 generations (data not shown).

Next, we compared the glucose homeostasis of control WT mice with that of *Ins1-luc* BAC transgenic mice. Every examination we tested, including blood glucose levels and insulin content in pancreatic islets, showed no difference between them, indicating that *Ins1-luc* BAC transgenic mice exhibit no abnormality in glucose homeostasis and therefore could be useful as β-cell-specific reporter mice for study of pancreatic islets (Figure S2A–F).

**Figure 3:**
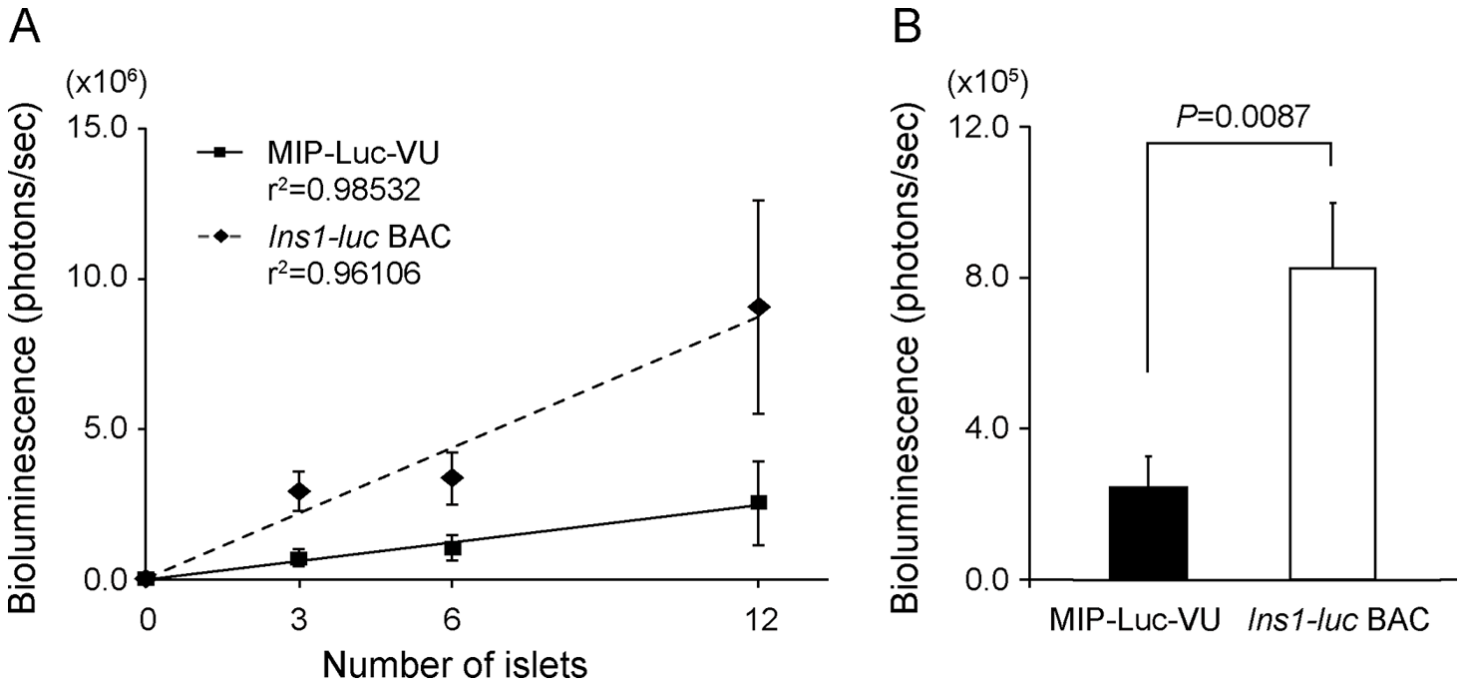
Bioluminescence intensity of isolated pancreatic islets in culture. (A) 3, 6, and 12 islets of similar size from MIP-Luc-VU mice (n = 4) and *Ins1-luc* BAC transgenic mice (n = 5) were individually placed in a 24-well plate, and bioluminescence imaging was performed immediately after addition of luciferin. (B) Comparison of bioluminescence intensity per islet in MIP-Luc mice (n = 6) and *Ins1-luc* BAC transgenic mice (n = 12).

To determine the relationship between the number of islets and the emission of bioluminescence, a variable number of equal-sized islets isolated from *Ins1-luc* BAC transgenic mice (n = 5) were placed individually in 24-well plates for 8 hours. The bioluminescence intensity emitted immediately after addition of luciferin to the culture media correlated positively with the number of islets (*R^2^* = 0.961) (Figure 3A). In addition, the bioluminescence emission from *Ins1-luc* BAC transgenic mice (8.13±1.71×10^5^ photons/sec; n = 12) was about 4-fold higher than that from MIP-Luc-VU mice (2.41±0.82×10^5^ photons/sec; n = 7; *P* = 0.0087) (Figure 3B), consistent with the *in vivo* result shown in Figure 2D.

**Figure 4:**
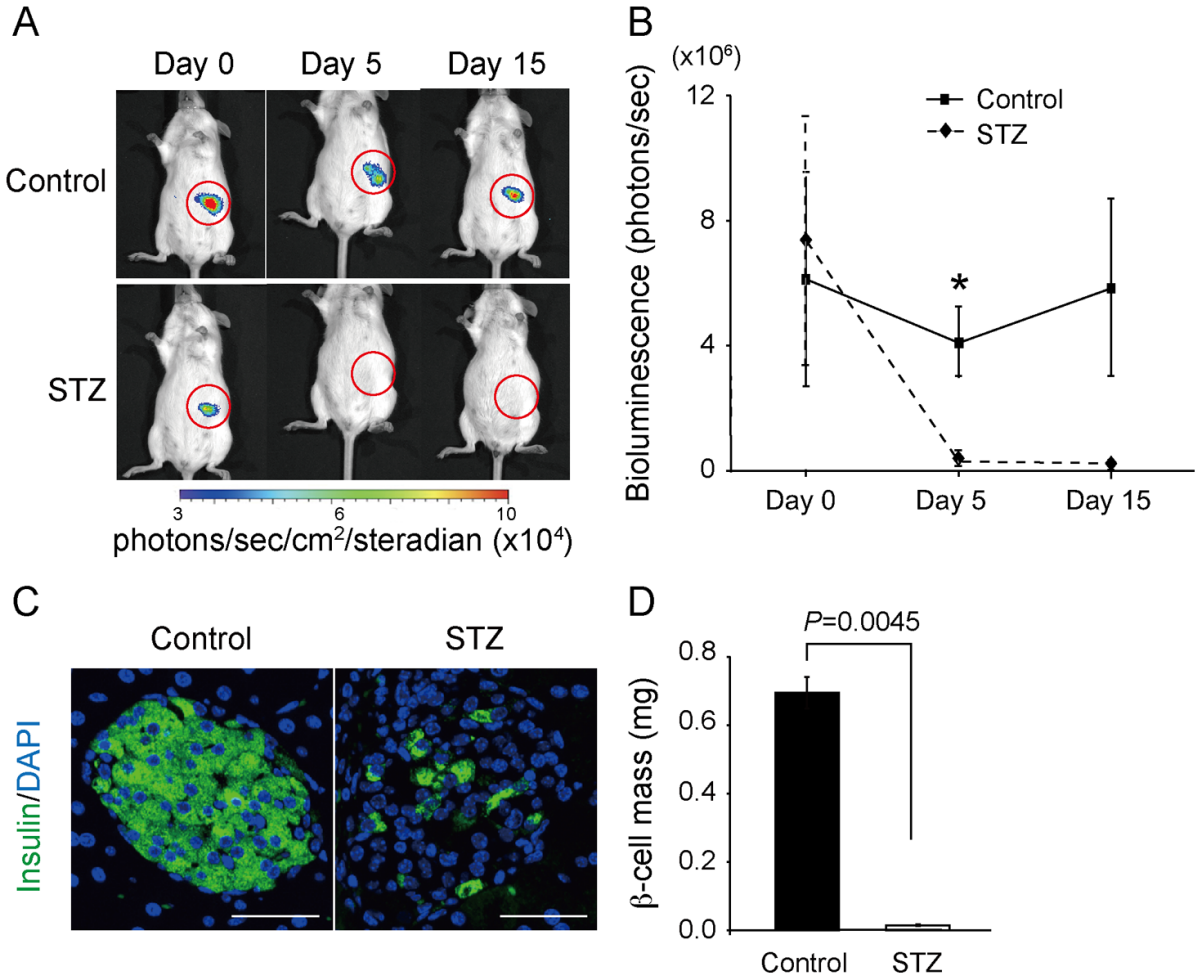
Bioluminescence images of streptozotocin (STZ) -treated *Ins1-luc* BAC transgenic mice. (A) Representative bioluminescence images of *Ins1-luc* BAC transgenic male mice before and after treatment with vehicle control or STZ. Circles indicate regions of interest. (B) Quantification of signal intensity in the vehicle control group (n = 5) and the STZ group (n = 5) at 0, 5, and 15 days after the injection. *P = 0.025. (C) Immunohistochemistry for anti-insulin antibody in the islets of *Ins1-luc* BAC transgenic mice treated with vehicle control or STZ. Scale bars: 50 µm. (D) β-cell mass in the vehicle control group (n = 3) and in the STZ-treated group (n = 4).

Next, we examined whether BLI of the mice could detect the loss of β-cell mass in an STZ-treated β-cell destruction model. On day 5 after the treatment, the luminescence in the pancreatic region of the STZ group (2.4±0.81×10^5^ photons/sec; n = 5) had dropped to undetectable background levels and was significantly reduced as compared with that of the control group (4.1±1.1×10^6^ photons/sec; n = 5; *P* = 0.025) in response to a decrease in β-cell mass (control: 0.70±0.05 mg, n = 4; STZ: 0.013±0.003 mg, n = 3; *P* = 0.0045) (Figure 4A–D).

**Figure 5:**
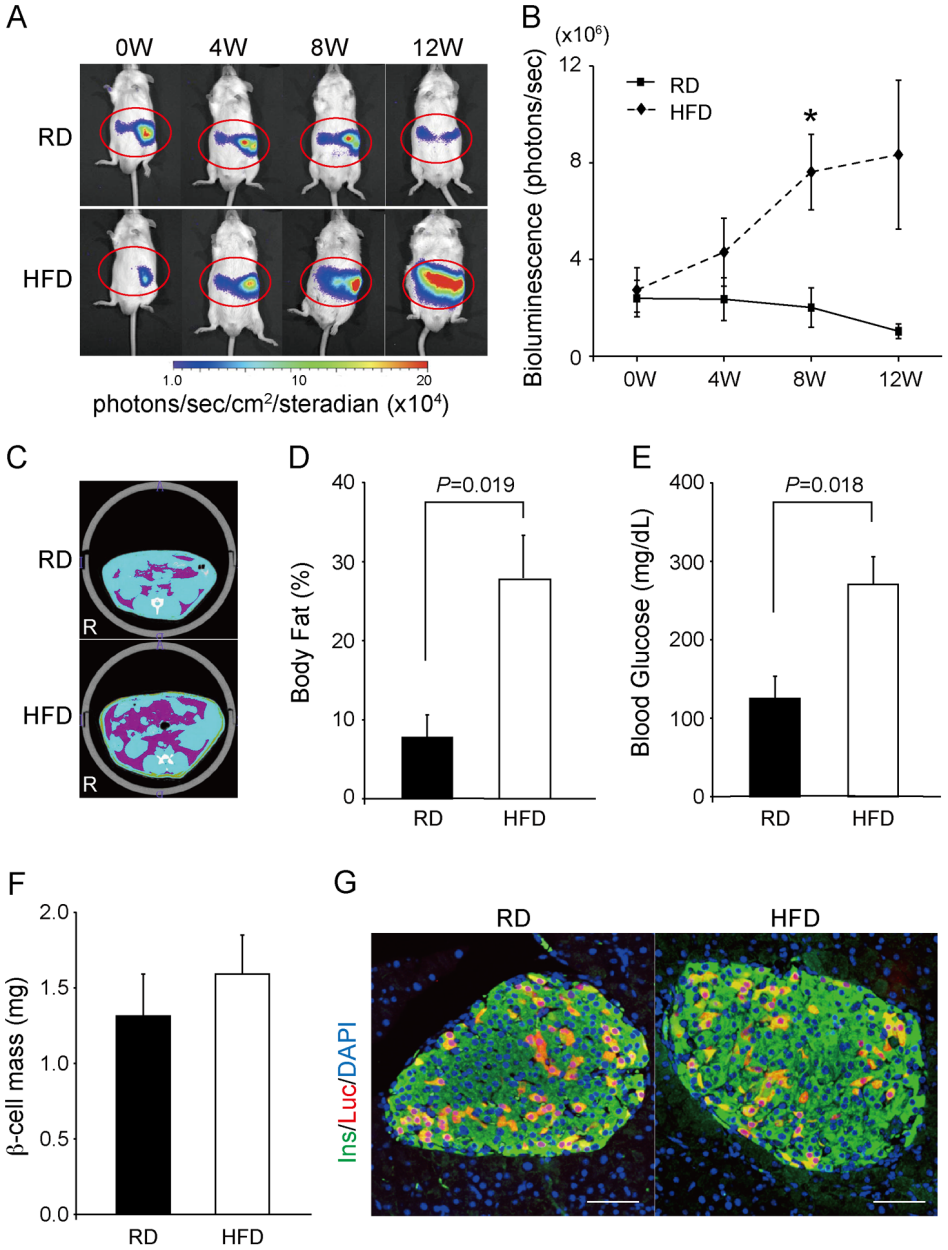
Bioluminescence images of *Ins1-luc* BAC transgenic mice fed a regular (RD) or a high-fat diet (HFD). (A) Representative images of mice fed a RD or an HFD for 12 weeks beginning at 6 weeks of age. (B) Quantification of bioluminescence intensity (RD: n = 4; HFD: n = 6). *P = 0.019. (C) Computed tomography (CT)-based body composition analysis. Representative CT images of mice treated with a RD or an HFD for 8 weeks. Purple, yellow, and blue areas represent visceral fat, subcutaneous fat, and lean mass, respectively. (D) Body fat percentage of mice treated with a RD (n = 4) or an HFD (n = 5) for 8 weeks. (E) Fasting blood glucose levels in the RD- (n = 4) or HFD- (n = 5) treated groups of mice for 8 weeks. (F) β-cell mass in the RD- (n = 4) and in the STZ- (n = 5) treated group. *P* = 0.49 (G) Immunohistochemistry for anti-insulin (Ins) and anti-luciferae (Luc) antibodies with diamidino-2-phenylindole (DAPI) for nuclear staining in the islets of RD- and HFD-fed *Ins1-luc* BAC transgenic mice Scale bars: 50 µm.

We also examined whether BLI could sense the increase in β-cell mass in HFD-fed mice. *Ins1-luc* BAC transgenic male mice fed either a RD (n = 4) or an HFD (n = 6) were imaged at 4, 8, and 12 weeks (Figure 5A). The bioluminescence intensity was significantly enhanced in the HFD-fed mice at 8 weeks (RD: 1.97±0.80×10^6^ photons/sec; HFD: 7.42±1.52×10^6^ photons/sec; *P* = 0.022) and accompanied by an increase in the body fat ratio (RD: 7.8±2.9%; HFD: 27.9±5.4%; *P* = 0.019) and in FBG levels (RD: 125±28 mg/dL; HFD: 271±35 mg/dL; *P* = 0.018) (Figure 5B–E). However, although the rate of increase in the signal after 8 weeks was substantially higher in the HFD-fed mice than in the RD-fed mice (379%), the rate of increase in β-cell mass differed slightly (121%; RD: 1.31±0.28 mg; HFD: 1.59±0.26 mg; *P* = 0.49) (Figure 5F). Immunohistochemical analysis of islets from HFD-fed mice showed a similar pattern to control islets in insulin and luciferase reactivity, so that we could not find a clear reason for the discrepancy in the rate of increase between the BLI signal and β-cell mass (Figure 5G) Interestingly, some mice, especially HFD-treated mice, developed dispersed luminescence from the central to right abdominal regions as shown in Figure 5, probably owing to the signal emission from the duodenum lobe that is ventrally covered by the liver and undetectable in MIP-Luc-VU mice.

**Figure 6:**
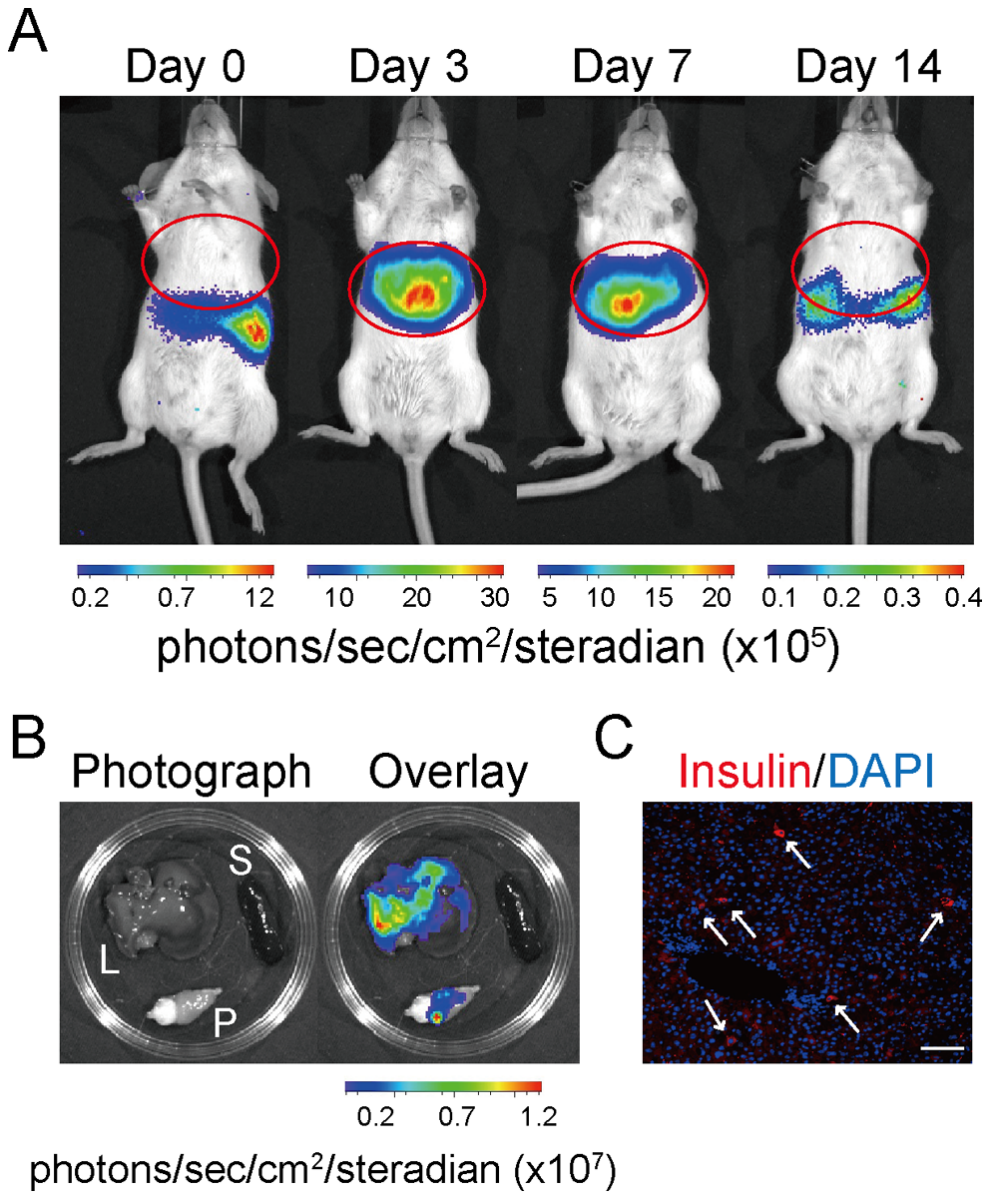
Bioluminescence images of *Ins1-luc* BAC transgenic mice with combined *Pdx1*, *NeuroD*, and *MafA* gene transfer in the liver. (A) Representative images of mice before (day 0) and after the gene transfer. Designated days indicate days after the infection. Each image is optimally adjusted using Living Image software because a huge difference in luminescence from the pancreas and the liver disables showing images with the same longitudinal photon scale. (B) Bioluminescence images of extracted organs from *Ins1-luc* BAC transgenic mice 3 days after infection. L, P, and S indicate the liver, pancreas, and spleen, respectively. (C) Immunohistochemistry for anti-insulin antibody of the liver from a WT mouse 3 days after infection. Scale bar: 50 µm. Arrows indicate insulin-positive cells.

Finally, we examined whether intrahepatic *insulin* gene activity is detected in *Ins1-luc* BAC transgenic mice by β-cell-related gene transfer. The *Pdx1*, *NeuroD*, and *MafA* genes were transferred by adenovirus vectors, and serial BLI was monitored before and after infection. This gene combination was selected according to a previous study [28]. The adenovirus-mediated gene transfer induced a BLI signal in the hepatic region for more than 1 week (Figure 6A). The signal was identified as originating from the liver by imaging of extracted tissues 3 days after infection and by immunohistochemical analysis using anti-insulin antibody (Figure 6B, C). Thus, these results indicate that *Ins1-luc* BAC transgenic mice could be used for monitoring intrahepatic *insulin* gene activity *in vivo*.

## Discussion

In this study, we generated and characterized novel *Ins1-luc* BAC transgenic mice in which β cells specifically express luciferase under *Ins1* locus control. Bioluminescence emission from the islets altered in response to both β-cell destruction and augmentation. Given also the absence of obvious phenotypic changes such as in islet morphology, β-cell insulin content, and secretory response by secretogens, these results indicate that these mice could be useful as a bioluminescence reporter line for β-cell studies.

We found that the bioluminescence intensity correlated well with the number of islets *in vitro*. However, although some damaged β cells remained after STZ treatment, luminescence from the area corresponding to the pancreas was not detectable. In addition, in the HFD model, the rates of increase of the BLI signal and the β-cell mass were hugely discrepant. These results suggest that *in vivo* BLI in the mice could provide only relative changes in β-cell mass but not absolute quantification under pathological conditions. In fact, the BLI intensity in another β-cell-specific reporter mouse has been shown to be affected by other factors, including chronic hyperglycemia, inflammatory mediators, and extrapancreatic fat mass [10].

As far as we know, the *Ins1-luc* BAC transgenic mouse is the first transgenic line containing a reporter driven by the *Ins1* locus. The approximate bioluminescence intensities emitted from the pancreatic islets of MIP-Luc mice developed by Park et al, of MIP-Luc-VU mice developed by Virostko et al, and of the *Ins1-luc* BAC transgenic mice developed here are 1.5×10^5^ photons/sec, 2.1×10^5^ photons/sec, and 7.9×10^5^ photons/sec, respectively [8], [9]. The strongest luciferase activities in *Ins1-luc* BAC transgenic mice depend on the cis- and trans-regulatory element(s) within the *RP23-181I21* and *luc2* gene as a reporter, which has been codon optimized for mammalian expression. However, the strong luminescence in *Ins1-luc* BAC transgenic mice might adversely affect individual and experimental variability. The luminescence intensity in *Ins1-luc* BAC transgenic mice showed relatively large variation among individuals, as shown in Figure 2B and 2D. This large variation probably arises because some mice develop adequately detectable luminescence from the pancreatic duodenum lobe that is covered ventrally by the liver. Therefore, the luminescence from the lobe generates a dispersed signal over the central to right abdominal regions especially in the HFD-fed mice (Figure 5A), resulting in individual and experimental differences.

Meanwhile, luciferase reporter expressions analyzed by immunohistochemistry are confined to approximately 10 to 20% of the total insulin-positive cells in the islets of both MIP-Luc-VU and *Ins1-luc* BAC transgenic mice (Figure 1D) [8]. MG132 treatment of *Ins1-luc* BAC transgenic islet cells revealed that proteasomal degradation of luciferase is mainly involved in the low coexpression of insulin and the reporter. Indeed, the high susceptibility of luciferase to ubiquitination confers its half-life of only about 3 hours in mammalian cells, whereas insulin secretory granules have a half-life of 3 to 5 days [29]–[31]. Besides proteasomal degradation of luciferase, β-cell heterogeneity regulating insulin expression and/or some posttranscriptional modifications observed in the mouse *Ins1* may also be involved in the levels of luciferase expression [32].

Adult pancreatic β cells were shown to be maintained by self-replication of resident β cells, not by differentiation from stem or progenitor cells [33]. Several factors driving β-cell proliferation have been identified, such as glucose, glucagon-like peptide-1 (GLP-1), connective tissue growth factor (CTGF), platelet-derived growth factor (PDGF), serotonin, and erythropoietin [34]–[39]. Stimulation of replication and maintenance of β-cell mass is considered to be an important therapeutic goal in diabetes. Alternatively, numerous attempts have been made to generate insulin-producing cells from other cell types. In this context, we found that β-cell-related gene transfer in hepatocytes of *Ins1-luc* BAC transgenic mice induced reporter gene activity for more than 1 week. This result indicates that *Ins1-luc* BAC transgenic mice could be useful for noninvasive monitoring of intrahepatic *insulin* gene activity *in vivo*. However, the reporter gene activity had disappeared by 14 days after the infection, indicating that the β-like cell conversion by *Pdx1*/*NeuroD*/*MafA* gene transfer was transient or incomplete. A recent study demonstrated that albumin-expressing hepatocytes could be directly converted into a neuronal cell lineage, implying that hepatocytes could be reprogrammed beyond different germ layers [40]. Indeed, it has been demonstrated that hepatocytes or hepatocyte precursor cells could be converted to insulin-producing cells using different protocols [41], [42]. However, a comprehensive method for comparing these protocols and determining the optimal conditions for β-cell generation has not been established. The combination of BLI and the transgenic mouse line described here provides readily quantifiable data to examine the efficiency of β-cell induction among different protocols.

## Supporting Information

Figure S1 **Proteasomal degradation is involved in the frequency of luciferase expression in β cells.** *Ins1-luc* BAC transgenic mice were euthanized at 8 weeks of age, and the pancreatic islets removed. Islets were treated with 10 µm MG132 (Wako, Osaka, Japan) in high-glucose DMEM (Invitrogen, Carlsbad, CA, USA) with 10% FBS. After 12 hours of incubation, tissues were fixed in 4% paraformaldehyde and embedded in paraffin. Tissue sections were incubated with guinea pig anti-insulin (Ins) antibody (Abcam, Cambridge, UK) and goat anti-luciferase (Luc) antibody (Promega, Madison, WI, USA) for 8 hours at 4°C following antigen retrieval. The antigens were visualized using appropriate secondary antibodies conjugated with alexa488 and alexa594 with nuclear staining using diamidino-2-phenylindole (DAPI) (Invitrogen, Carlsbad, CA, USA). Scale bars: 100 µm.
(PNG)

Figure S2 **Normal glucose tolerance, insulin secretion, and islet morphology in** ***Ins1-luc*** **BAC transgenic mice.** (A) Glucose tolerance tests after intraperitoneal loading with 2 g D-glucose/kg of WT (484±29 mg/dL, n = 3) and *Ins1-luc* BAC transgenic male mice (543±14 mg/dL, n = 3) after a 6-hour fast (*P* = 0.139). (B) Plasma insulin levels of WT (0.72±0.07 ng/mL, n = 3) and *Ins1-luc* BAC transgenic mice (0.79±0.21 ng/mL, n = 3) after intraperitoneal glucose injection (*P* = 0.78). (C) Plasma insulin levels of WT (1.08±0.22 ng/mL, n = 3) and *Ins1-luc* BAC transgenic mice (1.10±0.07 ng/mL, n = 3) after intraperitoneal arginine injection (*P* = 0.81). (D) Insulin content of WT (4W: 74.1±10.8 mg/g, n = 4, *P* = 0.15; 10W: 27.9±5.0 mg/g, n = 4, *P* = 0.19) and *Ins1-luc* BAC transgenic mice (4W: 96.7±8.3 mg/g, n = 4; 10W: 38.7±4.6 mg/g, n = 3) at 4 and 10 weeks of age (4W: *P* = 0.15; 10W: *P* = 0.19). (E) Glucose-stimulated insulin secretion (GSIS) from isolated islets of WT (1.7±0.35 ng/islet/hour; n = 5) and *Ins1-luc* BAC transgenic mice (2.1±0.41 ng/islet/hour; n = 5) at 8 weeks of age (*P* = 0.79). Values are expressed in nanograms of insulin/islet/hour. (F) Tissue sections stained with hematoxylin and eosin (HE) and immunostained with anti-insulin (Ins) antibody (Abcam), anti-glucagon (Glu) antibody (Linco Research, St. Charles, MO, USA), and diamidino-2-phenylindole (DAPI) (Invitrogen) of WT and *Ins1-luc* BAC transgenic mice at 8 weeks of age. Scale bars: 100 µm. Intraperitoneal glucose tolerance and arginine tolerance tests (IPGTTs and IPATTs) were performed after the mice had been fasted for 6 hours, as described previously (Zhang et al, 2005, Andrikopoulos et al, 2008, and Ayala J et al., 2010). Briefly, blood samples were collected from the retroorbital plexus at 0, 15, 30, 60, and 120 minutes after IP injection of glucose (2 mg/g of body weight). Plasma glucose levels were measured using a Drichem 3500 (Fujifilm, Tokyo, Japan). For insulin release, glucose (3 mg/g of body weight) or L-arginine (1 mg/g of body weight) was injected IP, and venous blood collected in heparinized tubes at 0, 2, 5, and 15 minutes. Pancreatic insulin was extracted by the acid-ethanol method as described previously (im Walde SS et al, 2002). Serum insulin levels and pancreatic insulin content were measured with a mouse insulin ELISA kit (Morinaga, Yokohama, Japan). To obtain pancreatic islets, pancreata were removed and the islets isolated by collagenase digestion using the previously described protocol (Lacy et al, 1967). These islets were washed and preincubated in 0.5% (wt/vol) bovine serum albumin-Krebs-Ringer HEPES-buffered saline in 2.8 mM glucose at 37°C in 5% CO_2_ for 30 minutes and then transferred to 0.5% (wt/vol) bovine serum albumin–Krebs-Ringer HEPES-buffered saline in 20 mM glucose. After being incubated at 37°C in 5% CO_2_ for 30 minutes, the supernatants were measured for insulin release as described above.
(PNG)
