# Supplementary figures and images for: Bioluminescence Imaging of β Cells and Intrahepatic Insulin Gene Activity under Normal and Pathological Conditions
Source: PLoS One. 2013 Apr 4;8(4):e60411. doi: 10.1371/journal.pone.0060411 (PMC3617225; doi:10.1371/journal.pone.0060411)

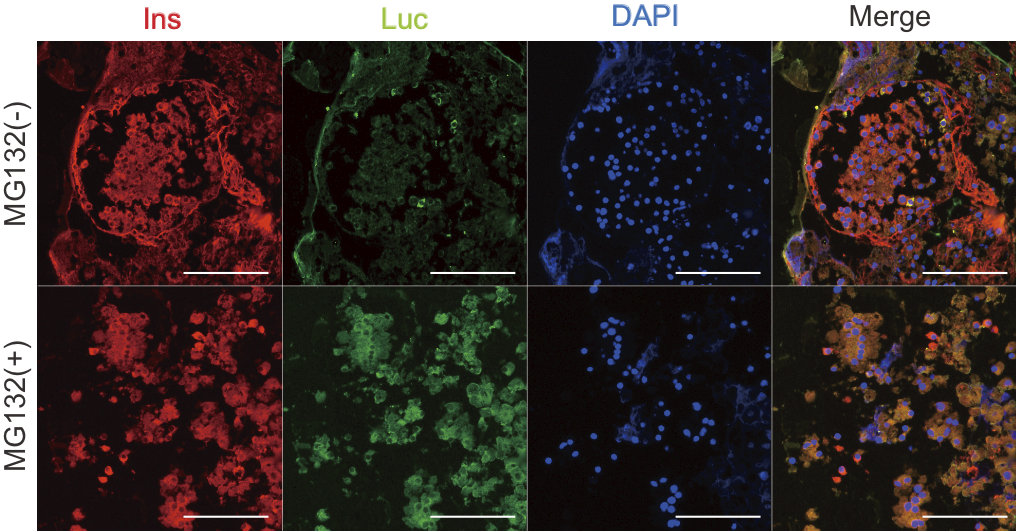

Supplement: Figure S1 — Proteasomal degradation is involved in the frequency of luciferase expression in β cells. Ins1-luc BAC transgenic mice were euthanized at 8 weeks of age, and the pancreatic islets removed. Islets were treated with 10 µm MG132 (Wako, Osaka, Japan) in high-glucose DMEM (Invitrogen, Carlsbad, CA, USA) with 10% FBS. After 12 hours of incubation, tissues were fixed in 4% paraformaldehyde and embedded in paraffin. Tissue sections were incubated with guinea pig anti-insulin (Ins) antibody (Abcam, Cambridge, UK) and goat anti-luciferase (Luc) antibody (Promega, Madison, WI, USA) for 8 hours at 4°C following antigen retrieval. The antigens were visualized using appropriate secondary antibodies conjugated with alexa488 and alexa594 with nuclear staining using diamidino-2-phenylindole (DAPI) (Invitrogen, Carlsbad, CA, USA). Scale bars: 100 µm. (PNG) [file pone.0060411.s001.png]

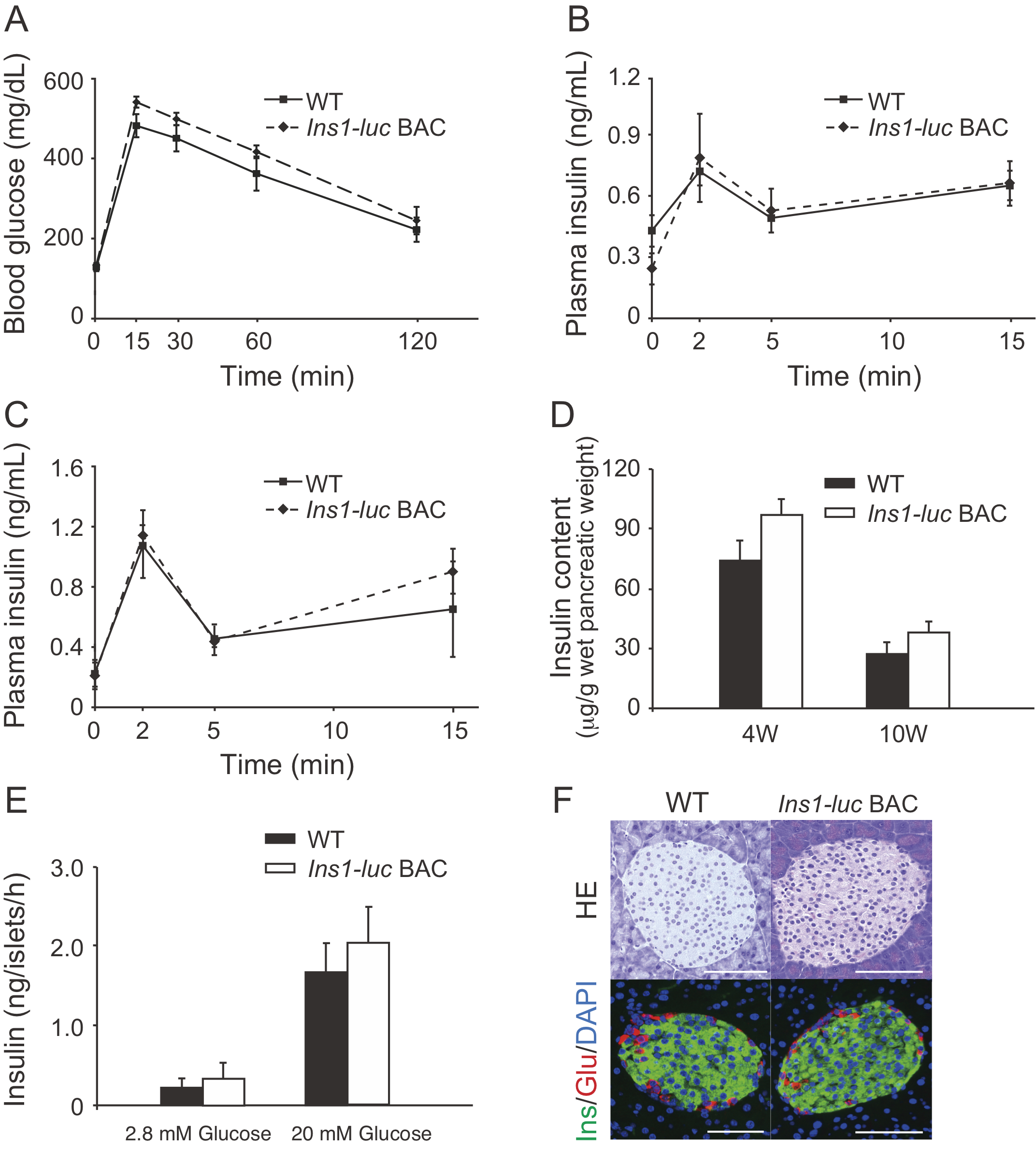

Supplement: Figure S2 — Normal glucose tolerance, insulin secretion, and islet morphology in Ins1-luc BAC transgenic mice. (A) Glucose tolerance tests after intraperitoneal loading with 2 g D-glucose/kg of WT (484±29 mg/dL, n = 3) and Ins1-luc BAC transgenic male mice (543±14 mg/dL, n = 3) after a 6-hour fast (P = 0.139). (B) Plasma insulin levels of WT (0.72±0.07 ng/mL, n = 3) and Ins1-luc BAC transgenic mice (0.79±0.21 ng/mL, n = 3) after intraperitoneal glucose injection (P = 0.78). (C) Plasma insulin levels of WT (1.08±0.22 ng/mL, n = 3) and Ins1-luc BAC transgenic mice (1.10±0.07 ng/mL, n = 3) after intraperitoneal arginine injection (P = 0.81). (D) Insulin content of WT (4W: 74.1±10.8 mg/g, n = 4, P = 0.15; 10W: 27.9±5.0 mg/g, n = 4, P = 0.19) and Ins1-luc BAC transgenic mice (4W: 96.7±8.3 mg/g, n = 4; 10W: 38.7±4.6 mg/g, n = 3) at 4 and 10 weeks of age (4W: P = 0.15; 10W: P = 0.19). (E) Glucose-stimulated insulin secretion (GSIS) from isolated islets of WT (1.7±0.35 ng/islet/hour; n = 5) and Ins1-luc BAC transgenic mice (2.1±0.41 ng/islet/hour; n = 5) at 8 weeks of age (P = 0.79). Values are expressed in nanograms of insulin/islet/hour. (F) Tissue sections stained with hematoxylin and eosin (HE) and immunostained with anti-insulin (Ins) antibody (Abcam), anti-glucagon (Glu) antibody (Linco Research, St. Charles, MO, USA), and diamidino-2-phenylindole (DAPI) (Invitrogen) of WT and Ins1-luc BAC transgenic mice at 8 weeks of age. Scale bars: 100 µm. Intraperitoneal glucose tolerance and arginine tolerance tests (IPGTTs and IPATTs) were performed after the mice had been fasted for 6 hours, as described previously (Zhang et al, 2005, Andrikopoulos et al, 2008, and Ayala J et al., 2010). Briefly, blood samples were collected from the retroorbital plexus at 0, 15, 30, 60, and 120 minutes after IP injection of glucose (2 mg/g of body weight). Plasma glucose levels were measured using a Drichem 3500 (Fujifilm, Tokyo, Japan). For insulin release, glucose (3 mg/g of body weight) or L-arginine [file pone.0060411.s002.png]
